# The 2024 ISCB Overton Prize Award—Dr Martin Steinegger

**DOI:** 10.1093/bioinformatics/btae288

**Published:** 2024-06-28

**Authors:** Mallory L Wiper

**Affiliations:** The International Society for Computational Biology


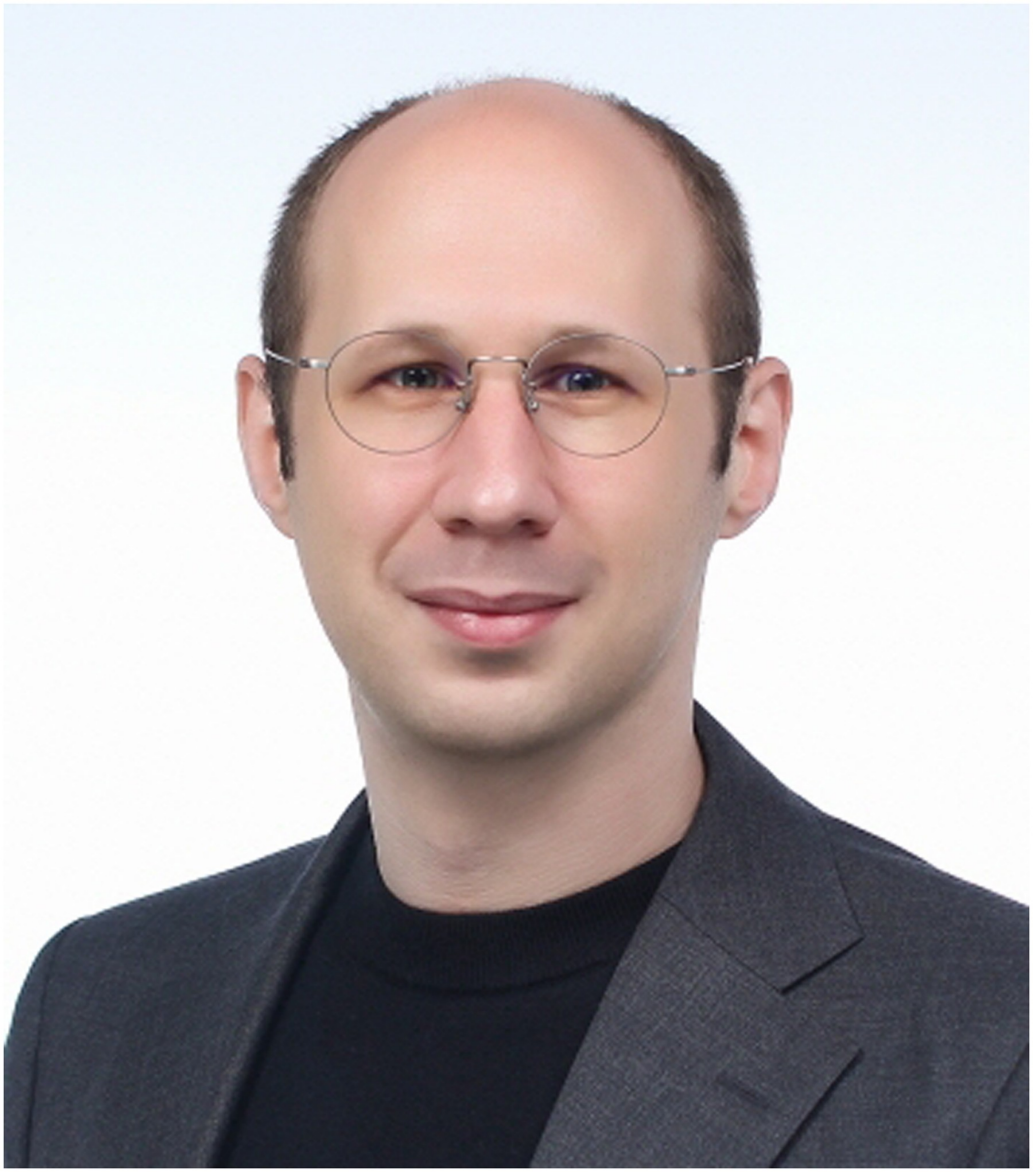


Each year, the Overton Prize is awarded to a scientist for their significant contributions to computational biology. This year, the International Society for Computational Biology (ISCB) has the pleasure of honoring Dr Martin Steinegger with this award at the 32nd Annual Intelligent Systems for Molecular Biology (ISMB) conference being held in Montreal, Quebec, Canada from July 12 to 16.

## Early inspirations

While Dr Steinegger is well entrenched in the field of computational biology these days, his path to this field wasn’t planned. In fact, when asked how he had become aware of computational biology, he said it was completely by accident!

Steinegger recalls his interest in computers being a foundational aspect of his academic journey. Specifically, it started when, as a child, he received a computer with a digital operating system and was very focused on getting games running—as any young child would be! This early experience helped fuel the spark of problem-solving.

Despite his interest in computers and technology, however, school was a different and more complicated story, and the path to university was anything but direct. Growing up in a small Bavarian town and being dyslexic, Steinegger was placed in an educational track that didn’t give him the option to pursue a university education. Instead, he was put on a path for job placement and was told that with his education, his options for the future were going to be limited. Instead of accepting limited options, Steinegger left the restrictive German education system behind to enroll in a technical school in Austria and later turned mandatory military service into civil service which allowed him to transfer to a professional school for business informatics. From there, he ended up working as an IT consultant, and though he enjoyed this position, it wasn’t the challenge he anticipated. Leveraging his professional schools’ education, and encouraged to do so by a former partner, Steinegger pursued a university education to find the challenge he was looking for.

## Discovery and interest in computational biology

Even with a strong technical background, abundant experience, and a desire to learn and to challenge himself, the decision to attend university was surprising to Steinegger’s father. He had expected that Steinegger would continue in the working world as he had done because that was a predictable, consistent, and stable career path, and he was concerned about the future his son would have following university. Nevertheless, Steinegger forged ahead! He does recall, however, that the beginning of his university career was fraught with feelings of inferiority due to his non-traditional academic career. Because of these feelings, he was determined to prove that he could handle the challenges of university. During the early semesters of his studies, he initiated ambitious independent research projects aimed at predicting the effects of mutations in the human proteome, alongside his close friend and co-worker, Dr Milot Mirdita.

The longer he attended university, his self-doubt waned, and the projects and challenges met became inspiration to explore and discover more instead of a means to prove himself. Eventually, Steinegger found himself working on a project that was investigating protein changes and whether such changes could be considered severe. The ability to answer this question was a *big* computational challenge and was a significant influence in setting him on a course to computational biology—because when there’s a challenge, he takes it on!

## Research and evolving pursuits

Despite the higher visibility projects like MMseqs2, Foldseek, and ColabFold, Steinegger is particularly partial to a project her surmounted during his PhD: Linclust, a clustering algorithm used to address the problem of slow clustering methods of metagenomic data that got even slower with the increasing size of datasets. With the relative speed of the Linclust algorithm, Steinegger was able to help address multiple questions that had previously been untouched, from clustering proteins in the billions to assembling genomes to detecting incorrectly labeled DNA in our biggest genomic databases.

When asked how the findings and outcomes from his work influenced his later research pursuits, Steinegger said that he knew the Linclust algorithm, or something similar, could be used in fields outside of protein study or computational biology. His hope is that researchers refrain from thinking only in terms of fields of study, effectively limiting themselves to a confined space, but instead think in terms of *opportunities*. Other fields have big questions waiting to be answered and a similar algorithm could be just the solution!

While he’s excited to answer as many big questions as possible, both in and outside of computational biology, currently, Dr Steinegger’s research interests remain focused on protein structures. Now more than ever, researchers have access to millions of high-quality predictions that could shed light on the structural universe. Protein structures can be viewed on an unprecedented scale; they can be analyzed in ways they were never able to be analyzed before. On a larger scale, there’s also the ability to more readily conduct comparative analyses and investigations of the natural world, exploring questions of how proteins and other machinery influence life on Earth.

## Training and mentoring new scientists

When it comes to training a new generation of computational biologists, Dr Steinegger’s personal training and mentorship experiences, as well as the shift to being a Principal Investigator (PI), have influenced his own methods of training and mentoring students.

Conducting research from the position of a PI has meant that, instead of only having to consider his own strengths in research, Steinegger now must consider the strengths and skillset of other people and has to be ready to adjust the questions being asked by young researchers to the skills they have and where he seems them growing.

Steinegger has taken an approach of optimistic support with new research projects for his students. That is, the potential pitfalls of research questions aren’t the initial focus. Instead, as a student’s research project is being built out, they start with the basic framework of an idea and consider how the question can be answered with the tools available, but also consider opportunities for new avenues of investigation.

The stance of making the basic idea work *before* considering pitfalls and complications comes from Steinegger’s own research experience, especially during his PhD. He vividly remembers having great, thought-provoking discussions with his advisor; discussions that challenged him to look at things differently, more simply. It was during this time that he really solidified his idea of “get it running first, then we can make it complicated.”

He is long since learned the lesson that perfectionism from the outset is not always possible or plausible. The drive for perfection sometimes stops forward momentum in research, and Steinegger tries to instill the idea in his own students that perfection *isn’t* the goal, but exploration and scientific discovery are!

## Reflections on the Overton Prize

When asked how it felt being named the 2024 Overton Prize winner, Steinegger summed it up by telling us that it “felt surreal” and that there are a lot of other people he saw as deserving of this award. He’s humbled to be winning this award and hopes to continue making strides to help answer the big questions in the field of computational biology.

